# Toward future diagnostics of Parkinson's disease: a perspective on multimodal motor assessment and personalized digital twins

**DOI:** 10.3389/fnagi.2026.1774177

**Published:** 2026-05-19

**Authors:** Júlia Rey Vilches, Sadasivan Puthusserypady, Bo Biering-Sørensen, Trine Hørmann Thomsen, Silvia Tolu

**Affiliations:** 1 Department of Electrical and Photonics Engineering, Technical University of Denmark, Kongens Lyngby, Denmark; 2 Department of Health Technology, Technical University of Denmark, Kongens Lyngby, Denmark; 3 Movement Disorder and Pain Research Center, Department of Neurology, Copenhagen University Hospital, Rigshospitalet, Glostrup, Denmark; 4 Department of Public Health, Faculty of Health and Medical Sciences, University of Copenhagen, Copenhagen, Denmark

**Keywords:** digital biomarkers, digital twins, early diagnosis, motor symptoms, multimodal data fusion, musculoskeletal modeling, Parkinson's disease, wearable sensors

## Abstract

The accurate diagnosis of Parkinson's disease (PD) remains challenging due to the heterogeneity of motor symptoms and the reliance on subjective clinical assessments. Although biomarkers such as dopaminergic imaging, fluid assays, and genetic testing can support diagnosis, their clinical utility is limited by cost, accessibility, and low sensitivity to early motor changes. Consequently, diagnosis depends largely on clinical observation, highlighting the need for more accurate, objective, and integrable tools to detect early motor symptoms. Advances in wearable sensing and computational modeling offer opportunities to address this gap. Multimodal approaches combining inertial measurement units (IMUs) and surface electromyography provide complementary information on movement and neuromuscular control. Moreover, IMU-derived kinematics can drive personalized musculoskeletal simulations, generating digital twins that estimate internal biomechanical quantities not directly observable from wearable data alone. This perspective aims to synthesize evidence and outline a clinically grounded framework for multimodal data fusion in PD, in which wearable- and model-derived features can be jointly analyzed. This approach enables the construction of a feature space capturing kinematic, neuromuscular, and biomechanical aspects of motor dysfunction, from which discriminative digital biomarkers may be derived. Rather than proposing new sensing or modeling technologies, the novelty lies in advancing a targeted integration strategy that evaluates which wearable modalities–and combinations–are most suitable for quantifying PD and driving musculoskeletal digital twins. Importantly, the framework is grounded in a protocol co-designed with movement-disorder specialists, ensuring clinically meaningful task selection, workflow compatibility, feasibility, and patient acceptability from the outset, supporting more interpretable and personalized diagnostic strategies for PD.

## 1 Introduction

### 1.1 Overview of Parkinson's disease

Parkinson's disease (PD) is often defined as a progressive neurodegenerative disorder characterized by degeneration of dopaminergic neurons alongside dysfunction of multiple neurotransmitter systems (Das et al., 2022; Titova et al., 2017). It is the fastest-growing neurological disorder worldwide (Ray and Okun, 2025), with prevalence projected to reach 12–17 million people by 2040 (Lancet, 2024; Luo et al., 2025), underscoring a growing clinical and socioeconomic challenge (Lancet, 2024; Ray and Okun, 2025).

PD manifests through motor and non-motor symptoms that progressively reduce independence, autonomy and quality of life (Meigal et al., 2013). Although bradykinesia (BK), tremor, and rigidity are core motor features, non-motor symptoms such as sleep disorders, depression and cognitive decline often precede motor onset. Moreover, PD presents with heterogeneous phenotypes, such as tremor-dominant, akinetic-rigid, and mixed forms, reflecting complex alterations in affected brain regions (Jankovic and Tan, 2020).

### 1.2 Emergence and progression of motor symptoms

Motor symptoms in PD progress gradually, often beginning with subtle asymmetries in movement amplitude and evolving to BK and rigidity. These typically appear only after 50–60% dopaminergic loss, when neurodegeneration is already advanced (Miller and O'Callaghan, 2015; Jankovic and Tan, 2020).

Early motor indicators–such as reduced arm swing, micrographia, diminished dexterity, and mild gait abnormalities–often remain undetected during standard clinical assessments. However, longitudinal studies show that minute abnormalities in tapping, rigidity, and mobility can be detected years before diagnosis (Maetzler et al., 2024). Importantly, wearable-based longitudinal tracking in newly diagnosed PD has shown that progression in selected motor features may be more detectable before antiparkinsonian treatment and become attenuated after treatment initiation, underscoring both the value of the early diagnostic window and the sensitivity demands placed on motor assessment tools (Brzezicki et al., 2023).

These findings reinforce the need for objective quantification of subtle motor deviations during disease onset. In the absence of curative therapies, early and accurate diagnosis remains the primary opportunity to influence clinical trajectory, guide treatment, and preserve quality of life.

### 1.3 Limitations of current clinical approaches: a need for objective tools

Diagnosis still relies primarily on expert visual assessment and patient-reported history (Postuma et al., 2015). Despite standardized tools such as the Movement Disorder Society Unified Parkinson's Disease Rating Scale (MDS-UPDRS) and Hoehn and Yahr (H&Y) staging, assessments remain subjective and prone to inter-rater variability (Spasojević et al., 2017; Sigcha et al., 2023). As a result, even specialists misdiagnose PD in up to 30% of cases, particularly in early or atypical presentations (Jankovic and Tan, 2020; Meigal et al., 2013; Guerra et al., 2023; Rábano-Suárez et al., 2025; Postuma et al., 2015). In primary care, where general practitioners are often the first point of contact, these limitations are amplified, as subtle early symptoms may be overlooked and referral delayed. Moreover, patient diaries commonly used to complement assessments lack ecological validity (Papapetropoulos, 2012).

Objective biomarkers such as dopaminergic imaging (DAT-SPECT, PET), fluid assays (e.g., α-synuclein), and genetic testing can support diagnosis but remain costly, invasive, or not widely accessible (Miller and O'Callaghan, 2015). Clinical levodopa trials, which assess motor improvement following dopaminergic therapy, are also considered a supportive diagnostic criterion and can aid differential diagnosis. However, none of these tools are standardized or suitable for routine screening or longitudinal monitoring.

These limitations have driven increasing interest in wearable sensing approaches (Bonato et al., 2024; Rábano-Suárez et al., 2025), which provide objective motor metrics and form the basis for **digital biomarkers**.

**Digital biomarker:**

Objective and quantifiable measures of physiological or behavioral function obtained from digital devices–such as wearables or sensors–that reflect health status, disease processes, or treatment responses (Coravos et al., 2019).

Despite extensive exploration of wearable technologies in PD, sensor choices are often optimized for symptom classification rather than holistic physiological interpretability or clinical integration potential. As a result, reported digital insights remain highly heterogeneous and fragmented across sensor modalities, movement tasks, acquisition protocols, and patient cohorts (Rábano-Suárez et al., 2025). Moreover, wearable sensing provides an incomplete view of motor dysfunction because internal biomechanic quantities (e.g., joint moments and muscle forces) are not directly observable from kinematics or surface recordings alone. Digital twins have emerged as a conceptual solution, enabling individualized, model-based representations of human physiology that are driven by, yet extend beyond, raw sensor data (Tang et al., 2024; Kamel Boulos and Zhang, 2021). In the context of movement disorders, musculoskeletal (MSK) modeling offers a particularly suitable foundation for such digital twins (Johnson and Saikia, 2024), however, existing applications remain limited to small studies and are dispersed across tasks and pathologies, with no unified strategy for integration with wearable sensing.

The novelty of this perspective lies not in proposing new sensing or modeling technologies, but in advancing a targeted integration strategy for PD assessment. It critically evaluates which wearable modalities–and combinations–are most suitable to quantify PD and drive MSK digital twins, and outlines how acquisition and analysis should be structured to maximize biomechanical insight and translational value. A central distinguishing element of the present framework is that workflow compatibility, patient acceptability, and clinical relevance are treated as design requirements from the outset through collaboration with movement-disorder specialists during protocol development, task selection, and feasibility assessment. This translational approach directly addresses a common failure mode in digital health research, in which technically promising systems are introduced with insufficient alignment with routine clinical practice and patient usability requirements. The framework therefore aims to support translatable physiologically grounded digital biomarkers that may contribute to earlier and more objective PD diagnosis.

## 2 Wearable sensing for PD: modality selection

PD causes characteristic alterations in movement execution and muscle activation, which can be partially quantified using wearable sensors. Over the past two decades, motion analysis has progressively shifted from complex laboratory-based systems toward lightweight wearable solutions (Bonato et al., 2024).

Current PD wearable research is primarily focused on IMU-based gait and tremor analysis at the wrist and lower limb, with limited sensor diversity and underrepresentation of neuromuscular and less-studied motor domains (Sigcha et al., 2023).

A wide range of wearable sensing modalities has been explored for PD assessment, including wearable systems such as plantar pressure insoles and smart garments, as well as laboratory-based Motion Capture (MoCap) systems with force plates and markerless approaches. While these approaches provide valuable information, particularly for gait analysis, they often increase setup complexity and reduce feasibility in routine clinical settings. In this perspective, IMUs and sEMG are selected based on a targeted integration rationale. IMUs provide segment-level kinematics that can quantify movement and, when combined with biomechanical modeling, enable the estimation of internal quantities such as ground reaction forces (GRFs) without additional instrumentation. sEMG complements this by capturing neuromuscular activation patterns and supporting the interpretation of model-derived outputs. Together, this combination supports a multimodal, minimal, portable, and clinically compatible sensing setup, while providing complementary information for PD characterization. Importantly, both modalities are also relevant for driving and supporting the validation of musculoskeletal digital twins (Section 3).

### 2.1 IMUs–capturing movement kinematics

IMUs can quantify kinematic parameters such as movement amplitude, speed, rhythm, symmetry, and variability, which relate directly to PD motor symptoms.

The number of sensors and their placement remain active areas of research. IMU-based PD assessment has been shown to be effective across sensor configurations ranging from full-body (e.g. MoCap system) to single- and reduced-sensor setups, enabling accurate diagnosis, differentiation from essential tremor (ET), task-based classification aligned with MDS-UPDRS and daily activities, and longitudinal tracking of early motor decline (Micó-Amigo et al., 2017; Lonini et al., 2018; Peres et al., 2021; Caramia et al., 2018; Ricci et al., 2019; Memar et al., 2018; Moon et al., 2020; Talitckii et al., 2020; Sotirakis et al., 2023).

However, the sensitivity of some IMU-derived kinematic features to disease progression has been shown to diminish following the introduction of antiparkinsonian medication (Brzezicki et al., 2023), further motivating the exploration of complementary modalities and model-derived variables that may capture aspects of motor symptoms not fully reflected in kinematic features alone.

### 2.2 sEMG–capturing neuromuscular activation

Surface electromyography (sEMG) provides insight into muscle activation patterns, motor unit recruitment and neuromuscular synchronization. In PD, sEMG reveals abnormalities in burst duration, amplitude modulation, activation timing, and antagonist co-activation, reflecting impaired neuromuscular control associated with rigidity, BK, and tremor (Meigal et al., 2013; Celicanin et al., 2024; Das et al., 2022; Vescio et al., 2021).

sEMG has been shown to quantify clinically relevant upper- and lower-limb PD impairments, correlating with MDS-UPDRS motor scores, distinguishing PD from healthy controls and ET, decomposing tremor components, and enabling machine learning (ML)-based classification and gait impairment detection (Pasmanasari and Pawitan, 2021; Robichaud et al., 2009; Kleinholdermann et al., 2021; Eftaxias et al., 2015; Kugler et al., 2013; Guo et al., 2020; Xing et al., 2022; Rey Vilches et al., 2026).

Time- and frequency-domain features remain dominant in sEMG-based studies, although wavelet and nonlinear methods combined with ML are increasingly explored to capture complex activation dynamics (Benatti et al., 2025; Chowdhury et al., 2013). Most studies emphasize upper-limb tremor or BK, with fewer targeting gait, balance, or rigidity.

### 2.3 Multimodal approaches: integrating wearable modalities for comprehensive assessment

Multimodal data fusion combining IMUs and sEMG has emerged as a promising strategy for characterizing PD motor symptoms. Early studies primarily focused on tremor and PD–ET discrimination (Hossen et al., 2010; Meigal et al., 2013), with more recent work extending multimodal assessment across resting and postural conditions to improve classification accuracy (Xing et al., 2022). Beyond tremor, multimodal approaches have demonstrated added value for assessing gait-related bradykinesia and freezing of gait (Kugler et al., 2013; Guo et al., 2020; Mazzetta et al., 2019), as well as upper-limb function during functional tasks with strong correlations to MDS-UPDRS outcomes (Spasojević et al., 2017).

However, these approaches typically combine modalities to improve predictive performance, without explicitly linking the signals through underlying physiological or biomechanical mechanisms. Progress is further constrained by heterogeneous study designs, limited standardization, and challenges in translating findings across tasks and disease stages. These broader limitations are discussed in Section 2.4.

### 2.4 Why wearable sensing remains insufficient for clinical integration

Despite advances in wearable sensing, most approaches remain focused on symptom classification rather than clinically interpretable biomarkers. Consequently, no clinically accepted digital biomarker for PD currently exists. Persistent challenges span methodological, practical, and physiological domains.

#### 2.4.1 Heterogeneity in study design and lack of standardized protocols

Methodological heterogeneity remains a major barrier to translation. Sensor type and placement, movement tasks, clinical endpoints, and analytical features vary widely across studies, often due to the absence of standardized and publicly available data collection protocols (Das et al., 2022; Di Biase et al., 2018). Many studies rely on custom movement tasks or partial clinical assessments targeting isolated motor domains, often focusing on either upper or lower limbs, limiting reproducibility and comparability across cohorts. Moreover, simplifying sensor setups too early risks overlooking whole-body coordination, a defining characteristic of PD motor dysfunction. A more systematic approach to protocol design and sensor selection is required to enable clinically meaningful outcomes.

#### 2.4.2 Limited personalization

Most wearable-based analyses focus on group-level trends rather than accommodating individual variability in anatomy or disease phenotype. This limits diagnostic specificity and reduces the potential for tailored assessments. Although wearable data can describe external motion patterns and surface muscle activity, they are inherently constrained in how much anatomical or biomechanical individuality they can represent.

#### 2.4.3 Practical and usability constraints

Even high-performance classification models often depend on rigid setups and lab-bound protocols that are impractical in routine clinical contexts (Di Biase et al., 2018). Usability remains a key limitation, as multi-sensor configurations often require expert placement and calibration in controlled environments, thereby limiting integration into routine clinical workflows (Di Biase et al., 2018; Das et al., 2022). Early collaboration between clinicians and engineers is therefore essential to align technical development with clinical feasibility.

#### 2.4.4 Physiological limitations of wearables: missing the biomechanical picture

Wearable sensors capture kinematics and surface muscle activation but cannot directly estimate internal biomechanical quantities such as joint moments, muscle forces, or GRFs–which are key to understanding motor impairment (Gamborg et al., 2023). Accessing these internal forces typically requires force plates or invasive instrumentation, which is not feasible in routine clinical practice. As a result, current wearable-only approaches provide an incomplete physiological picture, limiting their ability to capture the underlying biomechanics of PD movement.

## 3 Musculoskeletal modeling: bridging the gap between motion and biomechanics

Building on the limitations of wearable-only approaches discussed in Section 2.4, MSK modeling is introduced here as a necessary bridge between external sensor measurements and the mechanical processes they reflect. MSK models, simulate the human body as an articulated system of bones, joints, and muscles (Andersen, 2021). When driven by wearable-derived kinematics, they can estimate internal kinetic variables through inverse dynamics, enabling mechanistic interpretation of movement.

Traditionally, MoCap systems and force plates provided the inputs for MSK models (Andersen, 2021). Recent advances in wearable IMUs now allow these models to move beyond the laboratory and into real-world settings. While wearable-derived kinematics enable the estimation of internal kinetics, validating the physiological plausibility of the MSK-outputs, such as the resulting muscle activation patterns, remains essential. Although sEMG cannot directly measure muscle force, it remains an important reference for assessing whether the simulation framework meaningfully represents the movement being modeled (Lund et al., 2012; Melzner et al., 2022; Damsgaard et al., 2006; Fernandez et al., 2016; Andersen, 2021; di Biase et al., 2024). Validation using sEMG can assess whether the model reproduces expected activation trends across conditions and movements, as well as whether muscle activity profiles demonstrate comparable onset-offset timing, activation patterns across muscles, and envelope shape between simulated and recorded signals using different similarity metrics (Rodrigues et al., 2018; Melzner et al., 2022; Ashtiani et al., 2025; Tamilselvam et al., 2021). By contrast, direct agreement in absolute activation amplitude is inherently constrained by the nonlinear relationship between EMG and muscle force. Consequently, sEMG-based validation is most informative for evaluating timing, coordination, and relative activation behavior, and less reliable for confirming absolute force estimates (Section 4.1).

### 3.1 Biomechanical insights from MSK modeling in PD

MSK simulations are well established in movement science for estimating biomechanical variables (Alexander et al., 2021; Trinler et al., 2019; Kloeckner et al., 2023; Bertaux et al., 2022; Tamilselvam et al., 2021) and have also been applied in rehabilitation and clinical movement analysis for conditions such as stroke (Giarmatzis et al., 2022; Wang et al., 2022), cerebral palsy (Jing et al., 2023), and osteoarthritis (Goislard de Monsabert et al., 2014). In PD, however, their application is more recent and remains comparatively limited, although existing studies already suggest clear potential.

Current MSK-related work in PD falls into three broad categories: inverse modeling, EMG-informed neuromusculoskeletal modeling, and predictive simulation. Inverse modeling uses MoCap or markerless systems to estimate variables such as GRFs and joint moments during movement (Eltoukhy et al., 2017; Oh et al., 2020). EMG-informed models combine movement data with surface EMG to estimate internal muscle forces, coordination strategies, and joint torques that cannot be directly observed clinically (Romanato et al., 2022). Predictive simulations use simplified neural control frameworks to investigate mechanisms such as postural instability, altered tone, or delayed responses (Omura et al., 2023; Shanbhag et al., 2026). Together, these studies suggest that MSK modeling in PD remains an emerging but promising field. Notably, most existing work targets gait and postural stability, with limited attention to other motor domains central to PD assessment.

At present, most MSK models applied to PD rely on normative anatomy, generic tissue properties and muscle recruitment strategies. Therefore, they do not explicitly reproduce disease-specific phenomena such as rigidity-related tissue changes, pathological co-contraction, or altered neural drive, which may be more directly reflected in EMG signals. This is an important limitation. However, the immediate value of current models lies less in replicating PD pathology itself than in quantifying the biomechanical consequences of PD-specific movement behavior. Under normative assumptions, existing studies have identified measurable differences between PD and HC, including reduced braking and propulsion GRFs and altered lower-limb joint moments (Eltoukhy et al., 2017), lower force production, greater force variability, and altered torque patterns (Romanato et al., 2022; Mak et al., 2003).

These findings support a central conceptual point: when subject-specific models are driven by PD-specific movement data, altered kinematics, asymmetry, bradykinetic timing, reduced range of motion, and compensatory strategies can propagate through the mechanical system into distinct patterns of joint loading, GRF distribution, and muscle demand. Anatomical personalization remains an important next step, as accurate body dimensions and weight are needed for segment scaling and reliable internal estimates from patient-derived movements. Beyond anatomy, parameters such as passive stiffness, co-contraction tendencies, tendon properties, or neural delays may vary across PD phenotypes. Nevertheless, comparable heterogeneity is also present in healthy populations due to aging, lifestyle, training status, and genetics. The immediate objective is therefore not perfect physiological replication, but identification of robust, clinically meaningful biomechanical features that consistently reflect dysfunction despite inter-individual variability. Explicit encoding of pathological neuromuscular properties remains a longer-term modeling objective and is further discussed in Section 4.1.

Beyond biomechanical estimation, MSK modeling translates sparse wearable signals into a structured whole-body mechanical representation. IMUs provide localized signals and limited information on whole-body coordination, while sEMG reflects surface-level activation and altered neural drive but may miss deeper or synergistic muscles. Integrated within a simulation framework, MSK models can interpolate non-instrumented segments and estimate internal contributions such as joint loading, joint moments, muscle force redistribution, and balance mechanics. This is particularly relevant in PD, where motor symptoms may be linked to underlying mechanisms: reduced movement amplitude may reflect altered joint moments or muscle demands; slowness of movements may indicate reduced propulsion, abnormal joint power generation, or compensatory proximal strategies; and postural instability may involve impaired anticipatory control or asymmetric loading redistribution. This makes the MSK useful when the question is not only whether movement is altered, but why the observed kinematic pattern is altered.

Despite this potential, the existing research lines remain largely separate, with no unified multimodal framework integrating anatomical personalization, EMG information, robust kinetics, and clinically relevant tasks. This is where digital twin frameworks may offer substantial added value, and where the present work is positioned.

## 4 From wearables to musculoskeletal digital twins: a perspective on personalized biomechanical assessment in PD

Building on the barriers that currently limit translation of wearable research into clinical care, this perspective proposes an integrated strategy that combines multiple technological sources to support a more holistic understanding of motor impairment in PD. Importantly, the aim is to highlight how subtle impairments that emerge in early PD could be characterized and quantified with higher resolution through targeted multimodal integration. A wearable multimodal approach is discussed, involving two specific sensors—IMUs and sEMG—which have already been critically justified.

The multimodal strategy further incorporates outputs from musculoskeletal simulations driven by real movements captured through IMUs. These subject-specific simulation models are referred to here as MSK digital twins. In this study, a digital twin denotes a personalized virtual representation of the human body used to reproduce and analyze movement behavior. More broadly, digital twins integrate models and data to represent the properties, condition, and behavior of physical entities while enabling simulation of their real-world performance (Semanjski, 2023; Kshetri, 2021). However, unlike fully realized digital twins that operate as real-time, bidirectional systems with continuous updating, the present implementation is an offline musculoskeletal modeling framework personalized through subject-specific anthropometry and driven by wearable-derived kinematics. It should therefore be viewed as a foundational step toward future real-time digital twin systems.

In this way, biomechanical modeling serves as an interpretive layer linking observable movement patterns to underlying neuromechanical mechanisms. Integrating multimodal sensor data across clinically relevant and as-standardized-as-possible movement tasks may therefore enable more targeted, interpretable, and patient-specific assessment of a patient's condition.

**Figure 1:**
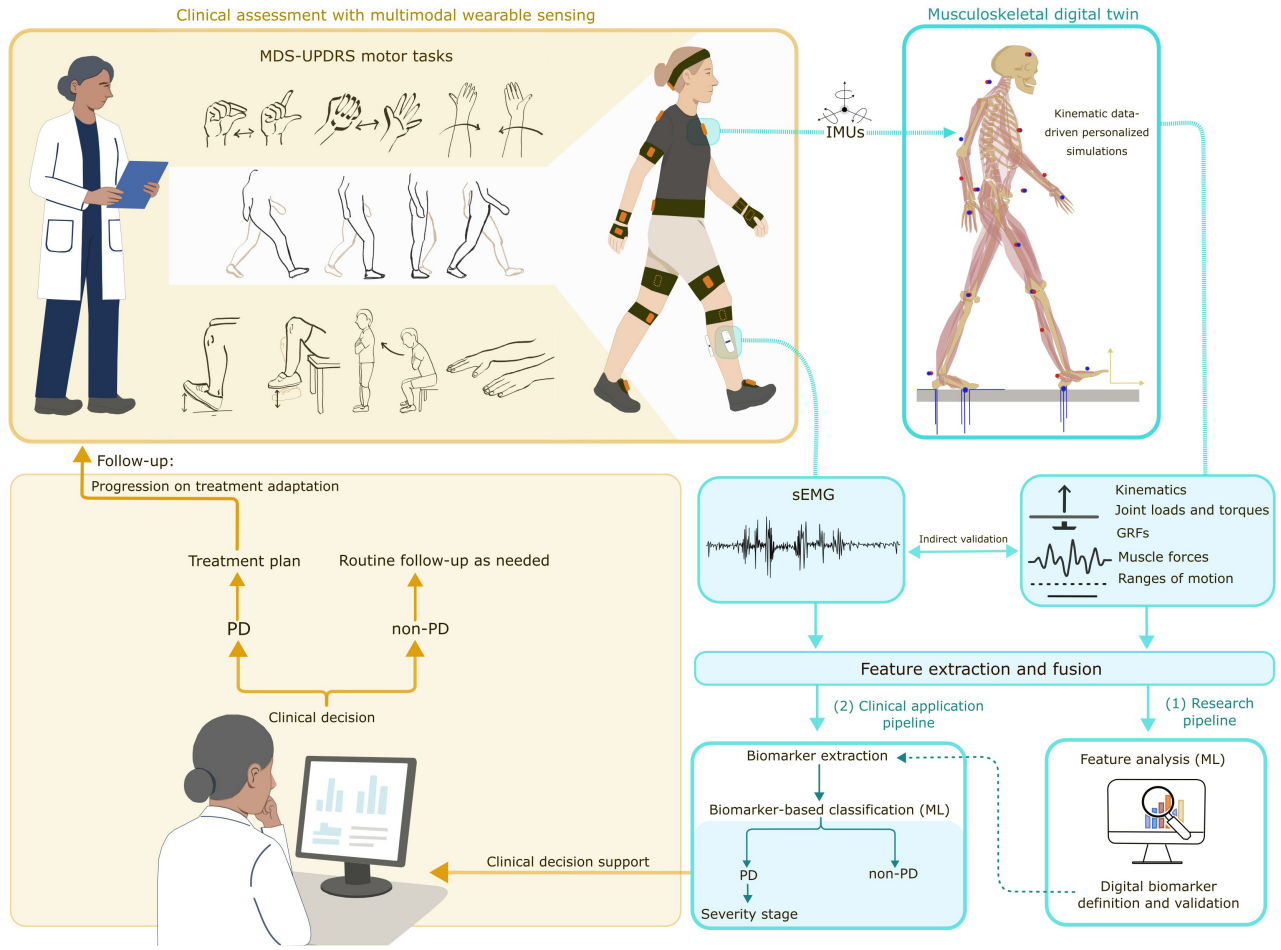
Multimodal motor assessment and personalized digital twin framework for PD: IMU and sEMG data collected during standardized motor tasks are integrated into a MSK digital twin to enable multimodal feature extraction and digital biomarker development for diagnosis and clinical decision support.

As illustrated in Figure 1, the framework follows a standardized multimodal acquisition pipeline in which synchronized IMU and sEMG recordings are collected during validated MDS-UPDRS motor tasks performed as part of routine clinical assessment, together with subject-specific anthropometric measurements (Vilches et al., 2025). IMUs provide segment-level kinematics that can further drive MSK simulations, enabling the model-based estimation of full-body movement, kinematics, kinetics, muscle forces, and coordination strategies that cannot be measured directly. In parallel, sEMG recordings provide an independent characterization of neuromuscular activation patterns associated with impaired motor control and rigidity, and serve as a physiological reference for assessing the plausibility of simulation-derived muscle-related outputs.

Personalized MSK digital twins are generated using an established simulation platform that integrates physics-based and data-driven modeling components. The physics-based layer represents musculoskeletal anatomy and biomechanical constraints, enabling the estimation of internal mechanical quantities and interactions not captured by wearable sensor data. The data-driven layer personalizes model parameters using subject-specific anthropometry and movement characteristics. IMU data inform the kinematic drivers of the simulations, whereas sEMG signals are not embedded within the model but instead are used as a physiological reference for neuromuscular interpretation and model consistency assessment.

Following biomechanical simulation, a dedicated data fusion layer integrates features derived from wearable sensing modalities (IMUs and sEMG) with simulation-derived outputs. This integrated representation forms the basis of the research pipeline, which focuses on identifying and validating features with discriminatory value for early PD detection.

Within this framework, ML techniques are used as structured analytical tools for feature selection, ranking, and exploratory classification (Rana et al., 2022; Tolu et al., 2023), enabling discriminative patterns and features to be traced back to their sensor origin and biomechanical relevance (Research pipeline, Figure 1). Feature selection and ranking strategies, including filter, wrapper, and embedded approaches (Wu et al., 2024), should be applied to manage the high-dimensional multimodal feature space and identify the most discriminative and stable feature subsets. Performance should be evaluated across multiple interpretable model types, such as regression models, support vector machines, and decision trees, using cross-validation to ensure robustness and generalizability of the identified features (Mei et al., 2021; Zakowicz et al., 2025).

It is worth noting that PD heterogeneity is not explicitly modeled within the ML pipeline, which identifies discriminative features at the group level. Personalization is instead introduced through subject-specific MSK modeling based on individual anthropometry and movement characteristics. In addition, recording each patient's predominant motor symptoms and medication status during data acquisition is important, as these factors can substantially influence motor feature expression. Such contextual information may also support downstream analysis by helping determine whether specific features are more strongly associated with tremor-dominant, rigidity-dominant, or other clinical presentations, while aiding interpretation of outliers and atypical cases.

Features identified and validated through the research pipeline are translated into a compact, interpretable subset of digital biomarkers for integration into clinical decision-support systems for PD diagnosis and severity staging (Clinical application pipeline, Figure 1).

Rather than replacing clinical judgment, this framework provides an objective, data-driven layer of evidence that complements traditional neurological examinations. Moreover, it may support more personalized treatment decisions, guide follow-up scheduling, and enable detailed longitudinal monitoring of disease progression.

The data acquisition protocol underlying the proposed framework was evaluated in a real-world clinical study involving 61 participants (31 individuals with PD and 30 HC), co-designed with movement-disorder specialists and assessed for feasibility and patient acceptance within routine clinical workflows (Vilches et al., 2025). The protocol enrolled participants with idiopathic PD across early-to-mid disease stages (H&Y 1—3), while excluding those with other neurological, musculoskeletal, or major orthopedic conditions that significantly affected motor function. The assessment comprised eight MDS-UPDRS motor tasks spanning upper- and lower-limb function, selected to ensure clinical relevance and feasibility within a standard outpatient visit. The data collection is not presented as proving the conceptual novelty, but as a demonstration of clinical integrability and serves as the empirical starting point for this perspective. Importantly, it addresses a persistent limitation in PD motor quantification research: the insufficient involvement of PD specialists in protocol design, task selection, and definition of clinically meaningful outcomes.

### 4.1 Methodological considerations and challenges

The proposed approach introduces methodological and translational challenges that must be critically considered.

- Wearable sensing and data acquisition:
  wearable-based assessments introduce uncertainties such as signal drift, soft-tissue artifacts, and variability across devices and configurations, which can propagate into biomechanical estimates and feature stability, particularly in multi-center studies. For sEMG specifically, reproducible electrode placement across sessions and operators represents an additional well-documented challenge. Variability in positioning can alter signal amplitude and frequency content, compromising longitudinal comparability and multi-operator consistency. Candidate mitigation strategies include standardized anatomical placement grids, and the use of pre-gelled electrode arrays that reduce operator-dependent variability (Hermens et al., 2000; Merletti et al., 2021).

These effects can be mitigated more broadly through rigorous calibration, standardized acquisition protocols, synchronized recording pipelines, and transparent reporting of preprocessing and modeling assumptions. Establishing minimal acquisition standards is therefore essential for reproducible digital biomarker development.

Another consideration relates to the data acquisition protocol itself. The MDS-UPDRS tasks used here provide a practical basis for multimodal acquisition because they were specifically designed to elicit clinically relevant PD motor symptoms during examination. They are therefore a logical starting point for capturing manifestations such as bradykinesia, tremor, gait impairment, and postural instability. However, as these tasks were developed for clinical rating rather than biomechanical sensing, their suitability for IMU-based kinematic analysis and inverse dynamics pipelines remains to be further validated.

- Musculoskeletal modeling assumptions and personalization limits:
  current models rely on generic assumptions regarding muscle recruitment strategies and tissue properties, reflecting normative rather than pathological neuromuscular behavior. Consequently, while inter-individual differences in movement dynamics can be captured, disease-specific alterations in muscle properties, ligament stiffness, or neuromuscular control are not yet explicitly modeled. Addressing this limitation remains a longer-term research challenge, particularly given the heterogeneity of PD and the substantial natural variability in musculoskeletal characteristics observed both in PD and healthy populations.

In addition, when IMU-derived kinematics are used to drive MSK simulations, further uncertainties may arise from orientation estimation errors, soft-tissue artifacts, and the absence of directly measured GRF. These sources of uncertainty may propagate through the inverse dynamics pipeline, leading to cascading effects on estimated joint kinetics and muscle forces.

Moreover, assessing the reliability of model estimates is necessary and requires experimental validation, for which sEMG is the most practical reference in wearable and clinical settings. Although surface recordings cannot capture deep muscles and do not map linearly to true muscle force, they remain valuable for verifying activation timing, coordination trends, and expected directional behavior. Agreement in these accessible muscles can therefore provide supportive–though not definitive–confidence in broader model outputs, including non-instrumented muscles within the same biomechanical framework. Although efforts have been made to estimate and validate these quantities, this remains an active area of research (Lund et al., 2011, 2012; Eltoukhy et al., 2017; Hicks et al., 2015).

- Digital biomarker extraction and data-driven analysis:
  extracting digital biomarkers from multimodal wearable and model-derived features introduces challenges beyond algorithmic performance. There is currently no consensus across PD studies regarding optimal features, sensor configurations, task selection, or analytical pipelines, making ML approaches highly dataset-dependent and limiting reproducibility and cross-study comparability (Mei et al., 2021; Halilaj et al., 2018). These challenges are amplified in multimodal settings, where the combination different modality-derived features can produce a high-dimensional feature space that increases sensitivity to feature selection choices and overfitting risk, a known concern in biomechanical and clinical ML applications with moderate sample sizes (Halilaj et al., 2018). Physiologically motivated feature pre-definition, cross-validation, and systematic evaluation of feature–model stability are therefore essential methodological safeguards, without which derived biomarkers risk being dataset-specific rather than generalizable.

Within ML, end-to-end deep learning approaches (e.g., neural networks) can learn feature representations directly from raw data. While powerful, these models often operate as black boxes, making their decision processes difficult to interpret and limiting their clinical applicability (Rudin et al., 2022). In contrast, the proposed framework adopts a feature-based approach, where ML operates directly on explicitly defined physiologically motivated features. These can be directly traced back to sensor modality, anatomical location, and clinically relevant symptoms. Accordingly, the research pipeline (Figure 1) emphasizes systematic evaluation of feature–model combinations that balance discriminative performance with transparency and physiological plausibility, enabling results to be meaningfully related to underlying biomechanical mechanisms.

Therefore, the approach prioritizes interpretable ML operating on structured inputs (e.g., regression models, support vector machines, decision trees), supporting transparent and clinically meaningful digital biomarker discovery.

- Clinical translation:
  clinical adoption requires validation across technical (sensor accuracy and modeling fidelity), analytical (alignment between wearable and biomechanical metrics), and clinical (association with severity, progression, and treatment response) levels (Taylor et al., 2020; Bakker et al., 2025). Multi-center and cross-country studies using harmonized protocols and shared outcome definitions are needed to ensured that derived digital biomarkers are robust, reproducible and acceptable across healthcare systems. Without such coordination, even physiologically grounded biomarkers risk remaining confined to isolated studies rather than achieving clinical adoption. Scalability is also a consideration. MSK simulations require subject-specific anthropometric measurements prior to setup and offline processing, with runtimes ranging from several minutes to tens of minutes per participant depending on model complexity and data volume. Cloud computing and batch-processing pipelines could facilitate larger-scale clinical deployment.

## 5 Outlook and future directions

Multimodal wearable sensing, particularly the combination of IMUs and sEMG, enables a more comprehensive characterization of PD motor dysfunction. IMUs quantify movement outcomes, while sEMG provides insight into the neuromuscular control that generates them. When interpreted through a musculoskeletal digital twin, these signals can be linked to internal biomechanical quantities such as joint loading, muscle coordination, and force redistribution, which are not accessible through wearable sensing alone.

In this context, personalized musculoskeletal digital twins should not be viewed as a replacement for wearable assessment, but as an interpretive and mechanistic extension of it. Their value lies in transforming externally measured movement abnormalities into physiologically grounded representations of how the body may be functioning internally. This creates the possibility of identifying digital biomarkers that are not only discriminative, but also interpretable, patient-specific, and more closely aligned with the biomechanical mechanisms underlying PD motor impairment.

Achieving clinical translation, however, will require standardized acquisition protocols, larger multi-center datasets with harmonized outcome definitions, transparent data processing pipelines, and multi-level validation spanning sensor accuracy, model fidelity, and clinical relevance. It will also require continued collaboration between engineers, modelers, and movement-disorder specialists to ensure that technical development remains aligned with real clinical needs, workflow constraints, and patient acceptability.

Taken together, these considerations define the central contribution of this perspective: to propose an integrative data-fusion framework in which multimodal wearable sensing and musculoskeletal digital twins are combined to improve the objectivity, interpretability, and clinical relevance of PD assessment. Rather than presenting a finalized diagnostic solution, this perspective outlines a translational pathway toward more personalized and biomechanically informed tools for earlier diagnosis and future longitudinal monitoring in Parkinson's disease.

## Data Availability

The original contributions presented in the study are included in the article, further inquiries can be directed to the corresponding author.
